# The Chicago College of Pharmacy

**Published:** 1867-06

**Authors:** 


### *The Chicago College of Pharmacy.*

It may be a matter of interest to many of our readers to learn that the Chicago College of Pharmacy is meeting with decided success. It numbers, on its roll of membership, the larger portion of the pharmaceutists of the city, with not a few from the country. In response to the circular from the Soliciting Committee, a number of donations have been received already, and many more are promised, so that there is good ground for the expectation that the College will soon be in the possession of an ample cabinet. Among the donations hitherto received, may be mentioned the extensive collection of chemicals, contributed by Messrs. Powers & Weightman, Philadelphia—a collection, not excelled, probably, by any other in the country; a large assortment of glassware, from Mr. Jeremiah Quinlan and Messrs. Hagerty Brothers, New York; specimens of plants from Southern Africa, from Messrs. Jas. C. Ayer & Co.; a collection of minerals from Mr. Chas. Oudeslness, Baltimore; numerous specimens of materia medica, from various parties, among them a large sample of the Calabar bean, now used by oculists for producing contraction of the pupil of the eye, donated by Dr. Hildreth; a specimen of the Cedron bean, by Dr. W. C. Lyman—a reputed cure for the bite of the scorpion; and many other specimens of rare drugs and pharmaceutical preparations.

The meetings are held every two weeks, in the afternoon and evening alternately. They have, thus far, been wéll attended, and a good degree of interest manifested by the members, several of whom have contributed papers on various subjects. A handsome certificate of membership has been engraved, and is now ready for distribution to members.

In accordance with a resolution passed at a recent meeting, non-resident apothecaries will be admitted to active membership on the same basis as those resident in the city, which, at present, is simply the payment of the annual dues—five dollars. It is earnestly hoped that many druggists throughout the North-west will feel sufficient interest to become members. By so doing, they will be encouraging a laudable and much needed enterprise. Any papers they may contribute, will be read and discussed at the meetings, and, if of sufficient general interest, will be published in this and other scientific journals. All communications should be directed to the Secretary, James W. Mill, 119 West Adams Street.

